# S1P Signalling Differentially Affects Migration of Peritoneal B Cell Populations In Vitro and Influences the Production of Intestinal IgA In Vivo

**DOI:** 10.3390/ijms19020391

**Published:** 2018-01-29

**Authors:** Annabel Kleinwort, Felix Lührs, Claus-Dieter Heidecke, Martin Lipp, Tobias Schulze

**Affiliations:** 1 Department of General Surgery, Visceral, Thoracic and Vascular Surgery, Surgery, Universitätsmedizin Greifswald, Greifswald 17475, Germany; annabel.kleinwort@stud.uni-greifswald.de (A.K.); andreasfelix.luehrs@stud.uni-greifswald.de (F.L.); heidecke@uni-greifswald.de (C.-D.H.); 2 Max-Delbrück-Centre for Molecular Medicine, Berlin 13125, Germany; lipp@berlin.de

**Keywords:** peritoneal B cells, B1 B cells, sphingosine-1-phosphate (S1P), mucosal immunity, lymphocyte trafficking

## Abstract

**Introduction:** Sphingosine-1-phosphate (S1P) regulates the migration of follicular B cells (B2 cells) and directs the positioning of Marginal zone B cells (MZ B cells) within the spleen. The function of S1P signalling in the third B cell lineage, B1 B cells, mainly present in the pleural and peritoneal cavity, has not yet been determined. **Methods:** S1P receptor expression was analysed in peritoneal B cells by real-time polymerase chain reaction (qPCR). The chemotactic response to S1P was studied in vitro. The role of S1P signalling was further explored in a s1p_4_^−/−^ mouse strain. **Results:** Peritoneal B cells expressed considerable amounts of the S1P receptors 1 and 4 (S1P_1_ and S1P_4_, respectively). S1P_1_ showed differential expression between the distinct peritoneal B cell lineages. While B2 cells showed no chemotactic response to S1P, B1 B cells showed a migration response to S1P. s1p_4_^−/−^ mice displayed significant alterations in the composition of peritoneal B cell populations, as well as a significant reduction of mucosal immunoglobulin A (IgA) in the gut. **Discussion:** S1P signalling influences peritoneal B1 B cell migration. S1P_4_ deficiency alters the composition of peritoneal B cell populations and reduces secretory IgA levels. These findings suggest that S1P signalling may be a target to modulate B cell function in inflammatory intestinal pathologies.

## 1. Introduction

Sphingolipids are important structural components of all eukaryotic cell membranes. The metabolites of complex sphingolipids (e.g., ceramide, sphingosine, and sphingosine-1-phosphate (S1P)) are also involved in the regulation of many important biological processes including cell survival, cell proliferation, cell differentiation, cell migration, and the rearrangement of the cytoskeleton (reviewed in [1]). As intracellular messengers, the balance between pro-apoptotic ceramide and sphingosine and anti-apoptotic S1P tightly regulates cell fate. This tightly regulated balance has been referred to as “sphingolipid rheostat” [2,3]. S1P also acts as an important extracellular messenger by binding to G-protein-coupled receptors on the cell membrane. Five distinct S1P binding receptors (S1P_1–5_) have been identified so far. Extracellular S1P concentration is highly controlled, with high concentrations in the blood and lymph and very low levels in most other tissues. This creates S1P gradients involved in the regulation of cell migration and other cellular processes in a wide variety of cells [4]. The crucial regulatory role of S1P has been shown in the immune system (reviewed in [5,6]), during angiogenesis [7], during carcinogenesis (reviewed in [8]), as well as in the cardiovascular system (reviewed in [9]), and notably during the development of arteriosclerosis [10].

There is a great heterogeneity in S1P receptor expression in cells of the immune system, both in the innate and the adaptive arm. In the innate immune system, macrophages express all S1P receptors. However, the expression of S1P_1_ and S1P_4_ appears to be regulated during macrophage polarisation towards the M1 or M2 phenotype, thus differentially influencing the biological effects of S1P signalling on the two functional states of macrophages [11]. Similarly, RNA of all five S1P receptors has been found in dendritic cells (DCs), and the expression of specific S1P receptors is regulated during DC maturation [12,13]. In the adaptive immune system, T cells were shown to predominantly express S1P_1_, S1P_4_, and S1P_2_ (under some conditions) [14,15,16]. Reports on S1P receptor expression on B cells are very heterogeneous, depending on the quantification method employed as well as the B cell population examined. Marginal zone B cells (MZ B cells) and follicular B cells (B2 cells) significantly differ in their expression of S1P_3_, but both express similar amounts of S1P_1_ and S1P_4_ [17]. Similar to the cells of the innate immune system, the S1P receptor profile on the adaptive immune cells has been shown to be non-static, but to vary according to the developmental stage and/or the activation status [16,17]. The relative expression level of S1P receptors on the cell surface determines the biological effect of S1P on the individual cell. In cells of the innate and adaptive immune system, S1P signalling has been shown to impact cytokine secretion, proliferation, and many other biological functions. The main biological regulatory function of S1P on immune cells occurs through the regulation of migration [12,15,18,19,20]. Migration is a key process in the immune system because it dictates the distribution of immune cells among primary and secondary lymphoid organs as well as effector sites. It also ensures the positioning of immune cells within different zones of lymphoid organs, thereby guaranteeing optimal physical interaction of immune cells.

In mice, B cells have three major subsets. Classical follicular B cells are part of the adaptive immune response and produce antigen/pathogen-specific antibodies after affinity maturation. The remaining two subsets—MZ B cells and peritoneal B1 cells—are heavily involved in the innate immune response. For this reason, they are referred to as innate B cells. B1 B cells differ from follicular B cells by their developmental origin, their surface marker expression, and their functional properties. B1 cells mainly secrete polyvalent immunoglobulin IgA antibody in a T cell-independent manner. They are thought to produce up the 40% of the serum IgA. Whether B1 cells contribute to the mucosal IgA in the intestine remains controversial. However, in mice, it has been shown in several experimental settings that up to 40% of intestinal IgA and IgA plasma cells are of B1 origin [21,22,23]. Furthermore, B1 cells are the major source of natural serum immunoglobulin M (IgM). After stimulation with lipopolysaccharide (LPS), peritoneal B1 B cells migrate to the spleen where they transform into IgM-secreting plasma cells [24,25]. Other than the generation of natural antibodies and plasma and mucosal IgA, B1 B cells exercise immuno-regulatory functions by influencing macrophage biology and T cell polarization [26,27]. B1 B cells are subdivided into B1a B and B1b B cells. While B1a B cells have been shown to intervene early in the immune response by the production of natural antibodies and their immuno-regulatory functions, B1b B cells are involved in the generation of long-lasting immunity independent of T cells [28,29]. Finally, B1a B cells appear to be the precursors of splenic innate response activator (IRA) B cells, which are involved in the control of polymicrobial sepsis [30]. These multiple functions involve the migration of peritoneal B cells to various secondary lymphoid and effector organs.

It has been previously shown that S1P signalling occurs both in B2 as well as in MZ B cells. S1P determines plasma cell homing towards bone marrow by facilitating plasma cell egress from the spleen [31]. In MZ B cells, S1P regulates the localisation of these cells in the marginal zone and, cooperatively with the chemokine CXCL13, controls the shuttling of these cells between the marginal zone and the white pulp [17]. Moreover, the expression of certain S1P receptors appears to be regulated during B cell differentiation [31,32]. S1P is also involved in the regulation of IgA plasmablast migration from Peyer’s patches (PP) to the lamina propria (Lp) of the gut [32].

While it has been clearly shown that in conventional follicular B2 B cells and in MZ B cells, S1P signalling controls several aspects of migration throughout the body and positioning within secondary lymphoid organs, much less is known about its role in peritoneal B1 B cells. In the present work, we explored the contribution of S1P signalling to peritoneal B1 B cell migration and function.

## 2. Results

### 2.1. Expression of S1P Receptor Subtypes on Peritoneal B Cell Subpopulations

**Figure 1:**
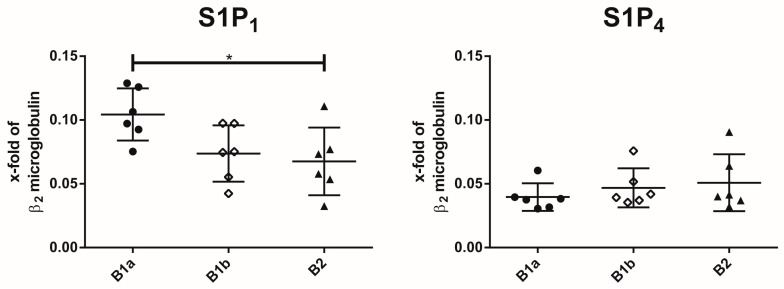
Expression of sphingosine-1-phosphate (S1P) receptor subtypes S1P_1_ and S1P_4_ in peritoneal B cell subpopulations. S1P receptor expression analysis was determined by real-time polymerase chain reaction (qPCR) on peritoneal B cell populations. Gene expression was normalized to β2-microglobulin. Values represent the mean and standard error of the mean of *n* = 6 animals per group. * *p* < 0.05.

The mRNA expression of S1P_1–5_ was assessed on all three peritoneal B cell subsets. Only S1P_1_ and S1P_4_ were expressed in all peritoneal B cell subsets (Figure 1). None of the remaining three S1P receptors could be detected in any of the peritoneal subpopulations (Supplementary Figure S1). The S1P_1_ receptor was differentially expressed in peritoneal B cell subpopulations, with B1a B cells showing the highest expression. In contrast, S1P_4_ expression levels were similar in all peritoneal B cell subpopulations.

### 2.2. S1P-Induced Chemotaxis Is Mediated Synergistically via S1P_1_ and S1P_4_

**Figure 2:**
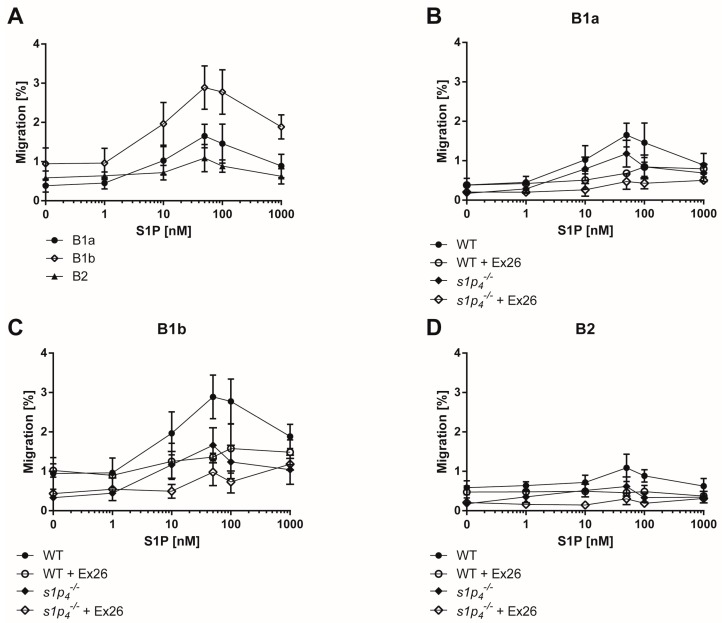
In vitro migration of peritoneal B cell subpopulations. In vitro chemotactic response to S1P was assessed in a transwell migration assay through a 5 µm membrane. (**A**) wild-type (WT) peritoneal cells; (**B**) Migration of B1a cells of WT or s1p_4_^−/−^ with or without Ex26, (**C**) Migration of B1b cells of WT or s1p_4_^−/−^ with or without Ex26, (**D**) Migration of B2 cells of WT or s1p_4_^−/−^ with or without Ex26. Values represent the mean and standard error of *n* = 6 (without Ex26) or *n* = 3 (with Ex26) per condition.

Since the control of cell migration is one of the most salient functions of S1P signalling in the immune system, we hypothesized that S1P regulates the migration of peritoneal B cells. We assessed the capacity of all three peritoneal B cell subpopulations to migrate along a S1P gradient in vitro. B1b B cells showed the highest chemotactic response to S1P, while the response of B2 B cells was markedly lower, close to background migration rates (Figure 2A). Next, we established whether this migration response was predominantly mediated by S1P_1_ or S1P_4_. Blockage of S1P_1_-mediated signalling by the specific S1P_1_ inhibitor Ex26 resulted in a clear reduction of the S1P-induced chemotactic response of B1a and B1b B cells (Figure 2B,C). However, both cell types preserved a small chemotactic response to S1P in the presence of Ex26. We next used s1p_4_^−/−^ cells to assess the role of S1P_4_ in S1P-induced chemotaxis in peritoneal B cell populations. Indeed, S1P_4_ deficiency reduced S1P-induced chemotaxis in B1a and B1b B cells, even though this reduction was less pronounced in B1a B cells than the reduction induced by Ex26 (Figure 2B,C). Finally, blockage of S1P_1_ by Ex26 in a s1p_4_^−/−^ background and S1P_4_-mediated signalling led to almost complete abolishment of S1P-induced chemotaxis in both B1a and B1b B cells. In peritoneal B2 cells, blockage of S1P_1_ and/or S1P_4_ did not affect the lack of chemotactic response to S1P (Figure 2D).

### 2.3. S1P_4_ Deficiency Induced Profound Changes in Peritoneal B Cell Populations

**Figure 3:**
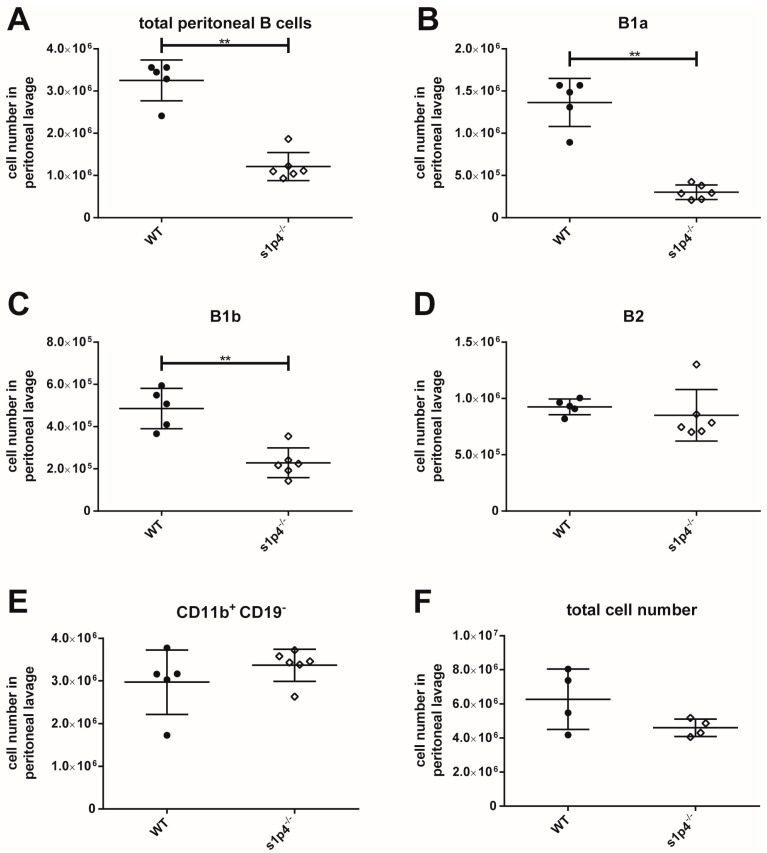
Composition of peritoneal B cell populations. Peritoneal lavage cells were counted and analysed by flow cytometry. Values represent the mean and standard error of *n* = 5 (WT) or *n* = 6 (s1p_4_^−/−^) animals per group. (**A**): Total peritoneal B cells; (**B**): peritoneal B1a B cells; (**C**): peritoneal B1b B cells; (**D**): peritoneal B2 B cells; (**E**): peritoneal CD11b^+^ CD19^-^ macrophages; (**F**): total peritoneal cell count. ** *p* < 0.01.

The functional S1P_1_ antagonist FTY720 has been shown to induce profound changes in peritoneal cell populations. However, the influence of S1P_4_-mediated S1P signalling on the composition of the peritoneal B cell population has not yet been assessed. Thus, we used s1p_4_^−/−^ mice to address this question. In s1p_4_^−/−^ animals, total peritoneal B cell numbers were significantly reduced (Figure 3A). Detailed analyses of the individual B cell populations revealed that this quantitative reduction concerned both B1a and B1b B cells (Figure 3B,C). In contrast, peritoneal B2 B cell numbers were similar in wild-type (WT) and s1p_4_^−/−^ animals (Figure 3D). Similarly, numbers of CD11b^+^ CD19^−^ peritoneal cells—which mainly represent macrophages—were identical in WT and s1p_4_^−/−^ animals (Figure 3E).

### 2.4. Influence of S1P_4_ Deficiency on Intestinal IgA Levels

**Figure 4:**
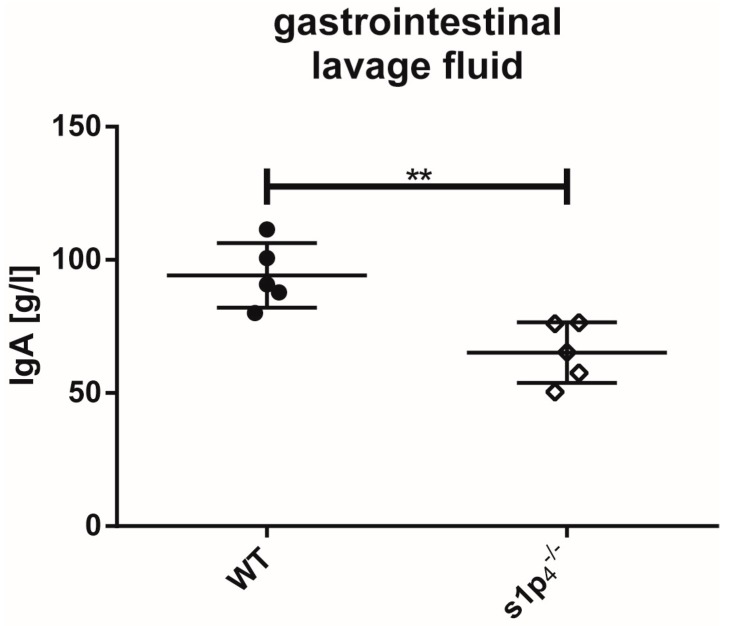
Immunoglobulin A (IgA) levels in gastrointestinal lavage fluid. IgA levels in gastrointestinal lavage fluid (GILF) were determined by enzyme-linked immunosorbent assay (ELISA) (*n* = 4–5 per group). ** *p* < 0.01.

Since peritoneal B1a B cells contribute to mucosal secretory IgA levels, we performed a comparative analysis of intestinal IgA levels in WT and s1p4^−/−^ mice. Interestingly, s1p4^−/−^ mice showed significantly reduced IgA levels in the gastrointestinal lavage fluid (GILF) of the small intestine compared to WT (WT: 94 ± 13 g/mL, s1p4^−/−^: 65 ± 12 g/mL (*p* < 0.01) (Figure 4).

## 3. Discussion

S1P-mediated signalling has been previously shown to profoundly affect the biology of follicular B2 B cells and MZ B cells [17,31,33]. A comprehensive analysis of the expression of S1P receptor subclasses in the third group of murine B cells—the B1 B cells, with their subpopulations B1a and B1b B cells—has not yet been performed. The present analysis by semi-quantitative qPCR establishes S1P_1_ and S1P_4_ as the only S1P receptors present on all three peritoneal B cell subsets. Interestingly, S1P_1_ expression in B1a B cells was significantly higher than in peritoneal B2 B cells, which differs from those reported by Kunisawa et al., who found similar S1P_1_ levels in the two cell types [34]. However, in their analysis, B1a B cells were not purified from B1b B cells, likely resulting in lower S1P_1_ levels for the total peritoneal B1 B cell population [34]. In peritoneal B2 B cells, we confirmed the lack of S1P_3_ expression reported by others [34]. Interestingly, the lack of S1P_3_ expression in peritoneal B2 B cells distinguishes these cells from splenic B2 B cells, which express moderate levels of S1P_3_ [17]. These results indicate that S1P_3_ expression is differentially regulated in B2 B cells in different localisations, potentially depending on their activation status, the local S1P concentration, or other cues of the local microenvironment. In order to assess whether the differences in the expression profiles of S1P receptor subtypes results in differential biological responses, we assessed their chemotactic response to a S1P gradient in vitro. Interestingly, we saw significant differences in the migratory response to S1P. B1b B cells were the most reactive peritoneal B cell population. As previously reported for splenic B2 cells, S1P did not induce migration in peritoneal B2 B cells. Using in vivo treatment with the functional S1P antagonist FTY720, Kunisawa et al. found similar reductions of peritoneal B1a, B1b, and B2 B cell numbers and concluded that the S1P mediated signalling exerted the same degree of influence on the different peritoneal B cell subpopulations [34]. However, our data clearly show that at least in vitro, S1P signalling induces a differential migratory response in the various peritoneal B cell populations. In order to evaluate the respective contribution of S1P_1_ and S1P_4_ to the S1P-induced chemotactic response, we performed further experiments using the S1P_1_-specific inhibitor Ex26 and S1P_4_ knock-out cells. Our results show that both receptors contribute additively to the migratory response induced by S1P. This is a particularly interesting finding, since we have shown previously that S1P_1_ and S1P_4_ may have an antagonistic influence on the migratory response to S1P in other cell types of the immune system [11,35]. These findings suggest that the intracellular signalling pathways employed by S1P_1_ and S1P_4_ might differ between B1 B cells and other cells of the immune system (e.g., T lymphocytes and bone marrow-derived macrophages).

As previously mentioned, treatment with FTY720—an S1P antagonist thought to predominantly act on S1P_1_-mediated signalling, has been shown to induce a dramatic reduction of peritoneal B cell populations by inhibiting B cell migration to the peritoneal cavity while enhancing B cell emigration [34]. We used s1p_4_^−/−^ animals to assess the influence of S1P_4_-mediated S1P signalling on the composition of the peritoneal B cell population. Our data show a clear reduction of B1a and B1b B cell numbers, while peritoneal B2 B cell numbers remained unchanged. Additionally, numbers of CD11b^+^ CD19^−^ peritoneal macrophages were not affected by the lack of S1P_4_. These data suggest that the presence of individual S1P receptor profiles on the various peritoneal B cell populations differentially affect migration not only in vitro but also in vivo, and that S1P_4_-mediated signalling selectively affects the B1 cell population in the peritoneal cavity. Further experimental work is required to elucidate the exact mechanism of this observation. Although it has been shown that inhibition of S1P_1_-mediated signalling with functional S1P_1_ antagonist FTY720 does not affect differentiation and apoptosis of peritoneal B cells [34], we cannot exclude that defective S1P4-mediated signalling affects the viability of peritoneal B1 B cells or their development from B1 cell precursors with our experimental design. S1P signalling influences cell migration in close interaction with chemokine-induced chemotaxis. Therefore, it is necessary to perform further in vitro and in vivo experiments to assess the influence of S1P signalling through S1P_1_ and S1P_4_ on the peritoneal B cell migration induced by CXCL12 and CXCL13 [36,37].

Impaired migration of peritoneal B cells and IgA plasmablasts results in reduced intestinal secretory IgA production [32,34]. In s1p_4_^−/−^ animals, secretory IgA levels in the small intestine were significantly reduced. We have previously reported that S1P_4_ deficiency results in normal faecal IgA levels under physiological conditions [35]. This apparent contradiction is explained by studies showing that faecal IgA content does not correlate with the amount of secretory IgA present in other segments of the gastrointestinal tract [38]. The reduced secretory IgA levels found in this study suggest that in s1p_4_^−/−^ animals, B1 B cell migration to this bowel segment is significantly impaired. Further experiments, including the transfer of S1P_4_-deficient peritoneal B cells, are required to verify this hypothesis. Under inflammatory conditions, S1P_4_ deficiency may result in an increased migration of B cells and IgA plasmablasts to the inflamed bowel sections, resulting in increased IgA levels, as we have detailed in a model of chemically-induced colitis [35]. Alterations in the local S1P gradients may be one explanation for this observation, since elevated S1P levels have been found in the colon tissue from colitic mice [39]. The exact mechanism leading to increased IgA levels in the faeces of s1p_4_^−/−^ animals with colitis remains to be determined.

Peritoneal B1 B cells are not only participating in the production of secretory IgA on mucosal surfaces, but also fulfil other important functions (e.g., production of natural IgM and immunoregulatory functions). We have previously reported only slightly reduced IgM plasma levels in s1p_4_^−/−^ mice; these differences did not reach statistical significance [35]. However, only a small percentage of the natural IgM is produced by peritoneal B cells, and the vast majority of the natural IgM is secreted by bone marrow and splenic B1 B cell populations [25,40]. It is necessary to assess the impact of S1P signalling on these B1 B cell populations. Peritoneal B1 B subsets also play a role as regulatory B cells. About 50% of peritoneal B1a and 24% of peritoneal B1b B cells showed the capacity for Interleukin (IL)-10 production [41]. Interestingly, long-term treatment with FTY720 in humans was reported to increase the frequency of IL-10^+^ and other regulatory B cells of the B2 lineage in the blood compartment [42]. Given the quantitative cellular changes in the peritoneal B1 B cell compartment in s1p_4_^−/−^ mice, it will be of interest to assess the impact of S1P_4_ deficiency on the regulatory function of peritoneal B1 B cells.

Proper migration of peritoneal B1 B cells to other immune compartments is implicated in various pathologies. The presence in the spleen of Granulocyte-macrophage colony-stimulating factor GM-CSF-secreting cells derived from peritoneal B1 B cells influences the clinical development of abdominal sepsis in mice [30]. The appropriate migration of peritoneal B1 B cells to the spleen after immunization was shown to regulate contact hypersensitivity. We have previously described the elevated response of s1p_4_^−/−^ mice in a model of contact hypersensitivity [30]. It is tempting to speculate that the altered migration of peritoneal B1 B cells in s1p_4_^−/−^ mice is causally implicated in the described effect.

In conclusion, S1P signalling represents a promising target to influence the biology of peritoneal B1 B cell populations in various pathological conditions where peritoneal B1 B cells play crucial roles in the pathogenesis (e.g., inflammatory bowel diseases, contact hypersensitivity, and sepsis) [28,43]. Since B1 B cells have very diverse biological functions requiring migration not only to the mucosal compartment but also to other immunological organs (e.g., the spleen), the question remains as to whether S1P-mediated signalling equally affects these migration processes [30,43]. Further experiments are required to characterize the impact of S1P-mediated signalling on the complex migration of peritoneal B1 B cells throughout various immune compartments in health and disease.

## 4. Materials and Methods

### 4.1. Mice

WT C57BL/6 mice were purchased from Charles River (Sulzfeld, Germany) and kept for at least two weeks before the experiments’ initiation to adapt to local conditions. s1p_4_^−/−^ mice on a C57BL/6J background were bred under specific pathogen-free (SPF) conditions in the Zentrale Service-und Forschungseinrichtung für Versuchstierkunde (ZSFV), Greifswald, Germany. Mice aged 10 to 14 weeks were used for all experiments. Antibody quantification in GILF was performed in the SPF animal facility of the Max-Delbrück-Center for Molecular Medicine, Berlin, Germany. All animal care practices and experimental procedures were performed in accordance with the German animal protection law (*TierSchuG*) and controlled by the veterinary government authority (Landesamt für Landwirtschaft, Lebensmittelsicherheit und Fischerei Mecklenburg-Vorpommern (LALLF-MV) and Landesamt für Gesundheit und Soziales Berlin (Project identification code: G0071/04, date of approval: 31 July 2004).

### 4.2. Cell Isolation from the Peritoneal Cavity

Murine peritoneal cavity cells (PerC) were obtained by peritoneal lavage. Peritoneal lavage was performed by intraperitoneal injection of 10 mL ice-cold PBS supplemented with either 2% foetal calf serum (Biochrom, Berlin, Germany) for RNA isolation or 0.5% fatty-acid-free bovine serum albumin (BSA, Sigma-Aldrich, St. Louis, MO, USA) for cell culture experiments involving later use of spinghosine-1-phosphate (S1P). Absolute cell numbers were determined using BD Trucount tubes (BD Biosciences, Becton Dickinson, Franklin Lakes, NJ, USA).

### 4.3. Flow Cytometry and Fluorescence-Activated Cell Sorting (FACS)

Nonspecific binding was blocked with an anti-FcγIII/II antibody (anti-CD16/32; BD Pharmingen, Heidelberg, Germany). The following antibodies and conjugates were used in the experiments in appropriate combinations: anti-CD5-phycoerytrhin (PE)/Cy7 (clone 53-7.3) (Biolegend, San Diego, CA, USA), anti-CD19-eFluor 660 (clone 1D3) (eBioscience, Thermo Fisher Scientific, Waltham, MA, USA), anti-CD23-PE (clone B3B4) (eBioscience), anti-CD3-FITC (clone 145-2c11), anti-CD11b-APC-eFluor 780 (clone M1/70) (eBioscience), anti-IgD-V450 (clone 11-26c.2a) (BD Biosciences), and anti-IgM-BV650 (clone R6-60.2) (BD Biosciences). For dead cell exclusion, cells were stained with 7-AAD Viability Staining Solution (Biolegend) before analysis. Stained cells were analysed on a BD LSR II Flow Cytometer (BD Biosciences) and evaluated with FlowJo software (Version 10, LLC, Ashland, OR, USA). The applied gating strategy is shown in Supplemental Figure S2. Fluorescence-activated cell sorting was performed on a FACSAria III cell sorter and gated in FACSDiva v8.0 (BD Biosciences). B1a B cells were identified as CD19^+^CD5^+^CD23^−^IgM^+^IgD^−^, B1b B cells as CD19^+^CD5^−^CD23^−^IgM^+^IgD^−^, and B2 B cells as CD19^+^CD5^−^CD23^+^IgM^low^IgD^+^.

### 4.4. RNA Isolation and qPCR

Fluorescence-activated cell sorted peritoneal B1a, B1b, and B2 B cells were loaded onto a QIAshredder spin column (QIAGEN, Hilden, Germany) and homogenized. For RNA isolation from the lysate, the RNeasy Mini Kit (QIAGEN) was used according to the manufacturer’s instructions. RNA concentration was measured by a Tecan Infinite N200 PRO (Tecan Austria GmbH, Gröding, Austria). Identical amounts of RNA (30 ng) were used for cDNA synthesis with the QuantiTect Reverse Transcription Kit (QIAGEN). Real-time RT-PCR for S1P1_1–5_ receptor expression was performed with the QuantiTect SYBR Green PCR Kit (QIAGEN) on an ABI Prism 7000 Sequence Detection System (SDS) (Applied Biosystems, Foster City, CA, USA). Analysis was done with the ABI Prism 7000 SDS software v1.1 (Applied Biosystems). Semi-quantitative gene expression was calculated according to the 2^−Δ*C*t^ method normalized to β_2_ microglobulin. The consistency of the internal control was verified by comparing 2^−ΔΔ*C*t^ values as described by Schmittgen et al. [44]. Primers were designed using Primer3 software (version 0.4.0, Whitehead Institute for Biomedical Research, Cambridge, MA, USA) and synthetized by BIOTEZ (Berlin, Germany). Primer sequences are listed in Table 1.

### 4.5. Transwell Migration Assay

Chemotaxis of individual peritoneal B cell populations in response to S1P was quantified using Transwell inserts (pore size 5 µm; Corning incorporated Costar) in a 24-well plate (Cellstar, Greiner Bio-One GmbH, Frickenhausen, Germany) according to the manufacturer’s recommendation. Absolute cell numbers of peritoneal B cell subsets were determined in an aliquot of the starting population by FACS analysis after staining with the antibody panels described above and using BD Trucount tubes (BD Biosciences). A total of 5 × 10^5^ peritoneal cells were added to the upper chamber in a volume of 100 µL. The lower chamber was filled with 600 µL migration medium containing different S1P concentrations (0–1000 nM). Cells were incubated for four hours at 37 °C and 5% CO_2_. Migrated cells collected in the lower chamber and absolute cell numbers were determined by FACS after staining with the antibody panels described above and using BD Trucount tubes (BD Biosciences). To specifically block S1P_1_, peritoneal lavage cells were pre-incubated with 1 µM Ex26 for 30 min at 37 °C and 5% CO_2_ [45]. One µM Ex26 was added to the migration medium of the upper and lower chamber of the Transwell migration assay. Migration (%) was calculated as follows: (Absolute cell number of lower chamber/absolute cell number of inserted cells in upper chamber) × 100.

### 4.6. Gastrointestinal Lavage

For gastrointestinal lavage (GIL), animals were sacrificed by cervical dislocation. The isolated small intestine was washed twice to discard any contaminating blood. The gut was then opened longitudinally. Both gut and intestinal content were mixed with 2 mL of GIL buffer (25 mM NaCl, 10 mM Na_2_SO_4_, 10 mM KCl, 20 mM NaHCO_3_, 50 mM ethylenediamine tetra-acetic acid (EDTA), 162 mg/mL polyethylene glycol (PEG), 1 mM phenylmethylsulfonyl fluoride (PMSF), and 0.1 mg/mL soybean trypsin inhibitor). After centrifugation, PMSF was added to the supernatant to reach a final concentration of 1 mM. The supernatant was stocked at −20 °C until further use.

### 4.7. Antibody Quantification

For determination of antibody concentrations in GILF and bronchoalveolar lavage fluid (BALF), the mouse MonoAB ID kit (AP, Zymed Laboratories, Burlingame, CA, USA) was used according to the instructions of the manufacturer.

### 4.8. Statistical Analysis

Statistical tests and graphs were performed with the GraphPad Prism software (Version 6.01, GraphPad Software, Inc., La Jolla, CA, USA). Groups were tested for Gaussian distribution with the Shapiro–Wilk test, and when normally distributed, statistical analysis was performed by *t*-test, one-way analysis of variance (ANOVA), or two-way ANOVA; otherwise, by Mann–Whitney-U test or Kruskal–Wallis test. *p*-values < 0.05 were considered to be significant. Significance in the graphs is labelled by * *p* < 0.05 or ** *p* < 0.01. Graphs show mean and standard error if normally distributed or median and interquartile ranges if not.

## Figures and Tables

**Table 1:** Primer sequences used for quantification of Sphingosine-1-phosphate (S1P) receptor subtypes and β_2_-microglobulin.

Primer	Sequence
S1P_1_ forward	5′-AAC TTT GCG AGT GAG CTG GT-3′
S1P_1_ reverse	5′-CTA GAG GGC GAG GTT GAG TG-3′
S1P_2_ forward	5′-ATA GAC CGA GCA CAG CCA AC-3′
S1P_2_ reverse	5′-GAG GTG GTC TCC TGC ATG TC-3′
S1P_3_ forward	5′-AAG CCT AGC GGG AGA GAA AC-3′
S1P_3_ reverse	5′-TCA GGG AAC AAT TGG GAG AG-3′
S1P_4_ forward	5′-GGA CTT CTC GGT CAC TCA GC-3′
S1P_4_ reverse	5′-GGC TTG CTG TCA TGT TCT CA-3′
S1P_5_ forward	5′-GGA GGG ACT CTC CTG GAT TC-3′
S1P_5_ reverse	5′-TTC CTC TGT AGC CAG CCA CT-3′
β_2_ microglobulin forward	5′-ATT CAC CCC CAC TGA GAC TG-3′
β_2_ microglobulin reverse	5′-GCT ATT TCT TTC TGC GTG CAT-3′

## Supplementary Materials

Supplementary materials can be found at http://www.mdpi.com/1422-0067/19/2/391/s1.

## Abbreviations

S1P	Sphingosine-1-P
BALF	Bronchoalveolar lavage fluid
MZ	Marginal zone
Ab	Antibody
DC	Dendritic cells
PP	Peyer’s patches
Lp	Lamina propria
WT	Wild-type
