# Supplementary material for: S1P Signalling Differentially Affects Migration of Peritoneal B Cell Populations In Vitro and Influences the Production of Intestinal IgA In Vivo
Source: Int J Mol Sci. 2018 Jan 29;19(2):391. doi: 10.3390/ijms19020391 (PMC5855613; doi:10.3390/ijms19020391)
Supplement: Supplementary file 1 [file ijms-19-00391-s001.docx]

Supplementary Materials: S1P signalling differentially affects migration of peritoneal B cell populations *in vitro* and influences the production of intestinal IgA *in vivo*

Annabel Kleinwort, Felix Lührs, Claus-Dieter Heidecke, Martin Lipp and Tobias Schulze


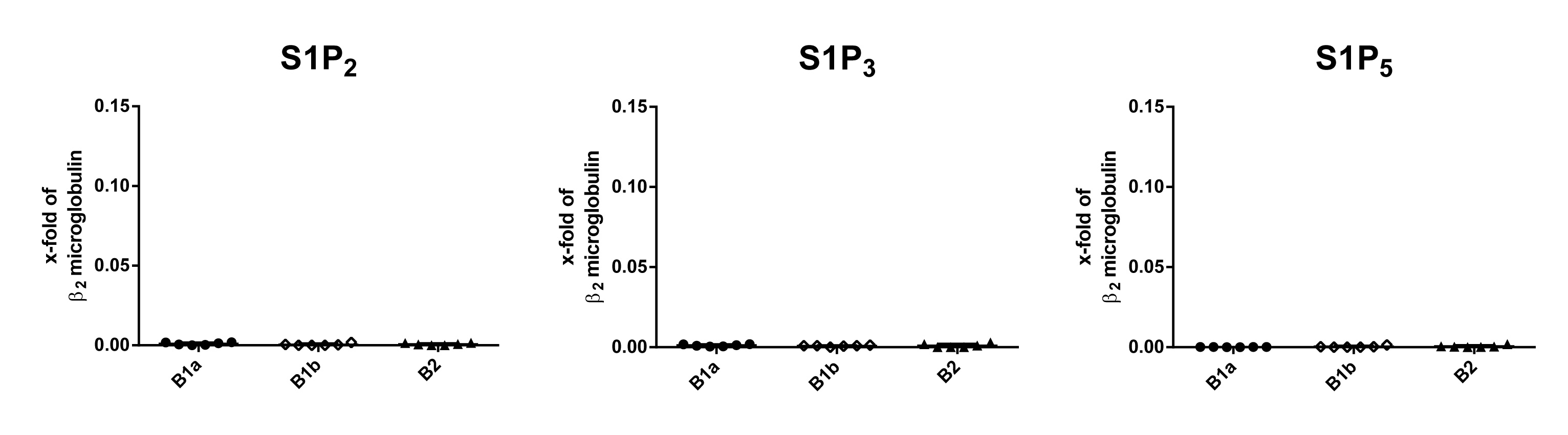


**Figure S1.** Expression of sphingosine-1-phosphate (S1P) receptor subtypes S1P_2_, S1P_3_ and S1P_5_ in peritoneal B cell subpopulations.


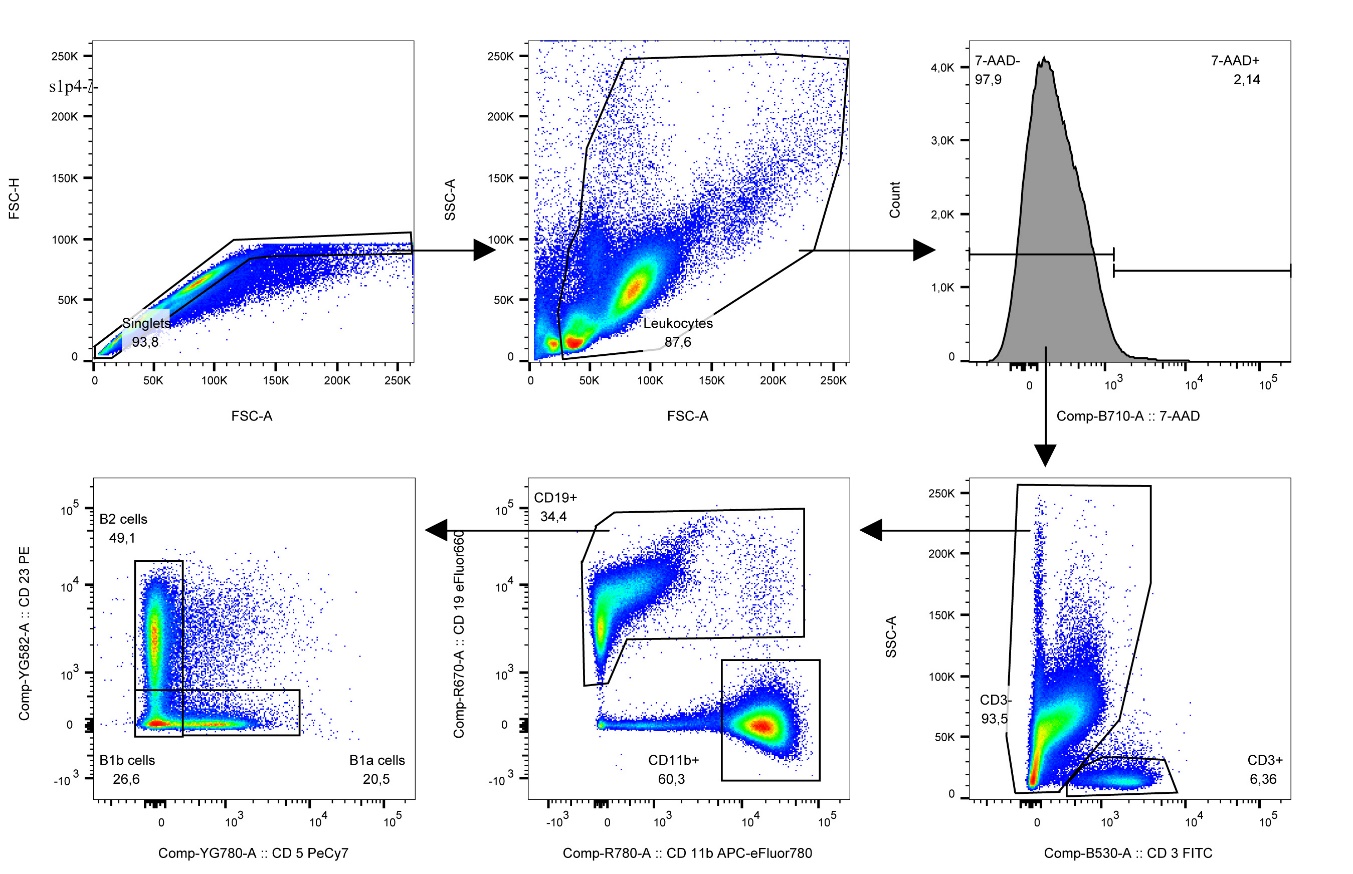


**Figure S2.** Gating strategy for identification of B1a B cells, B1b B cells and B2 B cells in the peritoneal cell population.
